# Essentials of hidradenitis suppurativa: a comprehensive review of diagnostic and treatment perspectives

**DOI:** 10.1097/MS9.0000000000002345

**Published:** 2024-07-01

**Authors:** Archana Pandey

**Affiliations:** Kathmandu University School of Medical Sciences, Dhulikhel Hospital, Dhulikhel, Nepal

**Keywords:** acne inversa, hidradenitis suppurativa diagnosis, hidradenitis suppurativa treatment, hidradenitis suppurativa

## Abstract

Hidradenitis suppurativa, or acne inversa, is a chronic inflammatory skin condition with recurrent inflammatory nodules, abscesses, subcutaneous tracts, and scars. This condition may cause severe psychological distress and reduce the quality of life for affected individuals. It is considered to have one of the most damaging effects on quality of life of any skin disorder as a result of the discomfort and foul-smelling discharge from these lesions. Although the pathophysiology of HS is still unclear, multiple factors, including lifestyle, genetic, and hormonal factors, have been associated with it. The pathogenesis of HS is very complex and has wide clinical manifestations; thus, it is quite challenging to manage and often requires the use of combination treatments that must be tailored according to disease severity and other patient-specific factors. Although lifestyle changes, weight loss, quitting smoking, topical treatments, and oral antibiotics are adequate for mild cases, the challenge for healthcare professionals is dealing with moderate-to-severe HS, which often does not respond well to traditional approaches. This literature review, consisting of an overview of the various assessment tools and therapy strategies available for the diagnosis and treatment of HS from published literature, aims to be a guide for practicing clinicians in dealing with the complexities associated with this disease.

## Introduction

Highlights

- Hidradenitis suppurativa (HS) is considered to have one of the most damaging effects on the quality of life of any skin disorder.
- A multidisciplinary team of dermatologists, surgeons, psychiatrists/mental health professionals, and pain specialists is required for the best management of patients with HS.
- Individualizing treatments according to the severity of the disease, treatment response, and the presence of co-morbidities is quite an important consideration in managing affected patients.
- Further research should focus on both expanding the current knowledge of the disease process and also on the evaluation of new treatments in clinical trials.

Hidradenitis suppurativa (HS) is a chronic inflammatory skin condition characterized by recurring inflammatory nodules, abscesses, and, subsequent to this, the occurrence of subcutaneous sinus tracts and scars^1^. It has a significant psychological and functional impact on patients due to the discomfort and odorous discharge from the lesions^2^. The global prevalence of HS is estimated to be 0.00033–4.1%, with a European-US population frequency of 0.7–1.2%^3^. The most common age of onset is between the ages of 20 and 40; however, new-onset HS has been documented in both younger and older individuals^4,5^. Because of poor mental health, lost employment, reduced intimacy, chronic pain, and substance use disorders, hidradenitis suppurativa has one of the most damaging effects on quality of life of any skin disorder^6–9^. These lesions appear in intertriginous regions and apocrine gland-rich areas, most often in the axillary, groin, perianal, perineal, and inframammary areas^10^. Multiple comorbidities, including metabolic syndrome, obesity, cardiovascular disease, chronic inflammatory bowel disease, and spondylarthritis, have been linked to HS^11,12^. Because of these issues, prompt diagnosis and efficient treatment are essential.

The pathophysiology of HS is still poorly understood. As a result, HS is a complicated condition with a difficult treatment. Although lifestyle changes, weight loss, quitting smoking, topical treatments, and oral antibiotics typically suffice for managing mild cases, dealing with moderate-to-severe hidradenitis suppurativa (HS), which often doesn’t respond well to traditional approaches, poses a significant challenge for healthcare professionals^13^.

This comprehensive review aims to offer valuable insights into the management of hidradenitis suppurativa (HS) by comparing assessment tools and treatment options. Our goal is to shed light on effective approaches that may benefit clinicians and researchers, thereby enhancing the care and understanding of this challenging condition.

## Methodology

Using keywords like ‘hidradenitis suppurativa’, ‘acne inversa’, ‘diagnosis’, ‘treatment’, ‘surgery’, and ‘medication therapy’, studies were searched in databases including PubMed, PubMed Central, and Google Scholar. This review includes case reports, case series, reviews, observational studies, randomized control trials, and meta-analyses discussing hidradenitis suppurativa. Only studies that have been published in English are included in this review. One hundred eleven papers are included in this evaluation after the studies were thoroughly extracted and analyzed.

### Pathophysiology

Although the etiology of HS is still unclear, significant advances have been achieved in recent studies^3^. In order to create effective treatments, it is imperative to further identify the various elements that contribute to HS. HS starts with follicular occlusion, which is likely due to infundibular keratosis and hyperplasia of the follicular epithelium, leading to the retention of secretions, rupture, and release of follicular contents^14^. Follicular content release in the dermis triggers an immune response that activates granulocytes, macrophages, plasma cells, and pro-inflammatory cytokines such as interleukins (IL-1, IL-17), tumor necrosis factor, and interferon, resulting in a vicious cycle of tissue destruction^15^. In later stages, this causes abscess formation, severe inflammation, sinus tract formation, and scarring^5^.

Multiple factors have been associated with HS, including lifestyle factors, genetic factors, and hormonal factors:

#### Lifestyle factors

Smoking, stress, and obesity are all lifestyle factors that contribute to the development of HS. A study discovered that people with HS were almost four times more likely to be smokers^16^. HS patients are also four times more likely to be obese than the general population, and BMI is positively correlated with disease burden^17^. It has also been suggested that psychological stress contributes to the onset and development of this illness. Stress can impair immune function, causing disease development or exacerbations^18^. Stress and worry can also lead to habits like smoking or binge eating, which can lead to the worsening of HS symptoms.

#### Genetic factors

In Western countries, HS normally appears as a sporadic form, but in 40% of cases, it arises as a family disorder^19^. Positive family histories are commonly reported by HS patients^20^, and autosomal dominant inheritance patterns in affected families have been identified^21^, which strongly suggests a genetic component to this condition. According to the genetic mutations linked to HS that have been discovered to date, HS can be inherited as a polygenic disorder brought on by abnormalities in genes controlling immune system function, ceramide synthesis, or epidermal proliferation, or as a monogenic trait caused by a defect in either the Notch signaling pathway or inflammasome function^20^. Chromosome 1p21.1–1q25.3 region has been linked to a mutation in the γ-secretase pathway in several families with HS patients^20,22^. These patients exhibit a severe phenotype of HS^20^. Mutations in the nicastrin (NCSTN) gene, presenilin 1 (PSEN 1), and presenilin enhancer (PSENEN) genes have all been linked to HS^19,20,23–25^.

#### Hormonal factors

Hormones are also considered a contributing factor in the pathogenesis of HS. Indicators of hormonal involvement include predominance in females, onset in puberty, and alterations in HS activity during times of changing hormones such as premenstrual periods, pregnancy, and menopause^26^. Activation of androgen receptors in the sebaceous gland causes increased sebum and inflammation, which can contribute to the blockage of hair follicles, ultimately leading to HS^26^. Although patients with HS have not been found to have greater than normal plasma levels of testosterone or 5-DHT, studies have shown that the use of anti-androgenic drugs has a beneficial response compared to antibiotic-based treatment alone^26^.

## Diagnosis

There have been several techniques reported for assessing patients with HS (Table 1).

**Table 1:** Assessment tools for hidradenitis suppurativa.

Assessment tool	Advantages	Disadvantages
1. Hurley^27–33^	Simple to use and provides rapid classification.	It is ineffective for assessing therapy responses.
2. The Hidradenitis Suppurativa Clinical Response (HiSCR)^34–36^	A valid, responsive, and meaningful endpoint for assessing the treatment effectiveness of HS in both clinical research and daily practice.	Its application is limited to evaluating treatment responses. HiSCR may lack sensitivity for milder cases due to its requirement of at least three abscesses and inflammatory nodules at baseline. Does not take draining tunnels into account in a dynamic manner, which can lead to an incomplete evaluation of the treatment response.
3. Modified Sartorius Score^2,37,38^	Dynamic scoring system that can detect changes in clinical disease severity.	Time-consuming, especially for patients with a wide-spread illness.
4. Physician Global Assessment Tool for HS (HS-PGA)^38–40^	Quick and simple assessment tool. Frequently used in clinical practice and clinical trials.	Largely dependent on the clinician’s capacity to identify essential lesions of HS.
5. The Hidradenitis Suppurativa Severity Index (HSSI)^38,39^	It is simple and rapid, utilizing subjective and objective criteria, and can assess disease severity without the need to differentiate between distinct elementary lesions. It also evaluates body surface area, drainage, and pain severity.	Lacks validation and lacks detail compared to the modified Sartorius score.
6. The International Hidradenitis Suppurativa Severity (IHS4) and IHS4-55^36,39,41–43^	It is a validated score that provides a dynamic assessment of HS severity in both clinical practice and the clinical trial setting.	Time-consuming due to the need for counting individual lesions, and individual lesion counts were demonstrated to differ between raters when compared to patients with milder forms of the disease.

HS, hidradenitis suppurativa.

### Hurley staging

**Figure 1:**
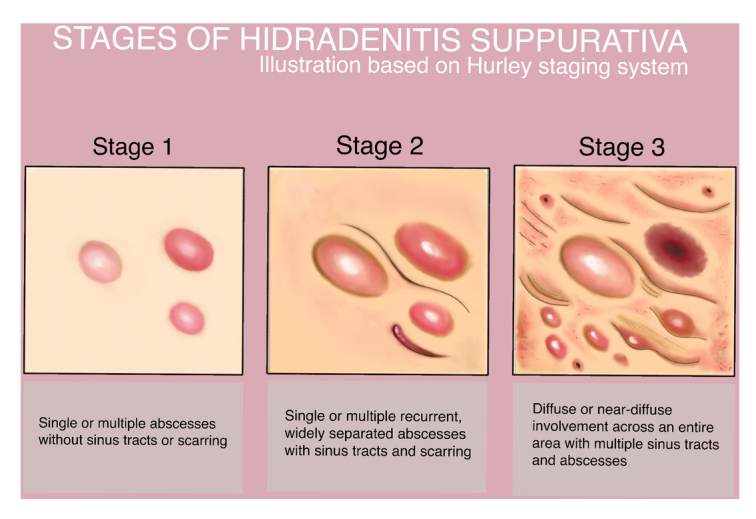
Stages of hidradenitis suppurativa according to Hurley staging system.

Hurley staging has been suggested in clinical settings due to its simplicity of use and rapid classification^27^. The Hurley scoring system is ineffective for assessing therapy response, as scarring may persist even after effective treatment^28^. Also, it is a static approach that is less appropriate for evaluating dynamic shifts in the severity of the illness^27^. Additionally, it does not measure the number of affected regions or evaluate disease activity or treatment response^29^. To address these limitations, the Refined Hurley staging has been developed^30^. The Hurley scoring system consists of three stages(Fig. 1)^30^:

Stage I: Single or multiple abscess formation without cicatrization or sinus tracts.
Stage II: Single or multiple recurrent abscesses, widely separated lesions with cicatrization and sinus tract.
Stage III: Diffuse or near-diffuse involvement across an entire area with multiple interconnected sinus tracts and abscesses.

Since clinical examination alone can underestimate HS and thus lead to insufficient treatment, some studies recommend the classification of Hurley stage based on ultrasonography, as ultrasound could detect early alterations that are unnoticeable clinically^31–33^.

### HiSCR

The Hidradenitis Suppurativa Clinical Response (HiSCR) has increased sensitivity to identify treatment effects and might be a helpful tool in clinical practice and research studies to assess the success of HS therapy^34^. This score does not indicate a patient’s severity at a certain time, but rather whether or not the patient improved between two time points, such as after receiving therapy. According to HiSCR, responders are those who obtain at least a 50% reduction in abscess and nodule count (AN-count) without increasing the number of abscesses or draining tunnels compared to the baseline^35^. HiSCR is a valid, responsive, and a meaningful endpoint for assessing the treatment effectiveness of HS in both clinical research and daily practice^35^. HiSCR was designed and validated for patients with at least three total abscesses and inflammatory nodules at baseline, limiting its sensitivity in detecting changes in milder forms of the disease^34^. Additionally, HiSCR does not dynamically include draining tunnels, potentially limiting its ability to fully assess the impact of anti-inflammatory therapy^36^.

### Modified sartorius score

This test counts individual nodules and fistulas in seven anatomical locations and measures the greatest distance between two lesions of the same type in each region^2^. This test is dynamic, meaning it is able to detect changes in clinical disease severity between visits^37^. However, calculating according to this scoring system is time-consuming, especially for patients with extensive disease^38^.

### HS-PGA

The Physician Global Assessment Tool for HS (HS-PGA) categorizes patients into six severity levels based on the quantity of abscesses, draining fistulas, inflammatory nodules, and non-inflammatory nodules^39,40^. Although HS-PGA is a quick and simple assessment tool, its effectiveness is largely dependent on the clinician’s capacity to identify HS essential lesions^38^.

### HSSI

The Hidradenitis Suppurativa Severity Index (HSSI) consists of five components: the number of sites affected, the body surface area (BSA), the number of inflammatory lesions, the number of dressing changes reflecting the quantity of drainage, and a visual analog pain scale^39^. This score uses both subjective and objective criteria to assess illness severity^39^. Furthermore, it may be estimated without separating between distinct elementary lesions, making it simple and rapid to use^39^. However, this test lacks validation and is not as detailed as the modified Sartorius score^38^.

### IHS4 and IHS4-55

The International Hidradenitis Suppurativa Severity (IHS4) is based on the number of inflammatory nodules, abscesses, and draining tunnels^41^.

IHS4-55, a revised scoring system, was created and approved in 2022. Patients are divided into two groups by IHS4-55: responders and non-responders. Patients were categorized as responders if their IHS4 score decreased by at least 55% over the course of two visits; non-responders were the remaining patients. Because of its increased inclusivity, IHS4-55 is a useful supplement to current scoring systems, enabling a more thorough evaluation of patients with HS^39,42^.

They rely on counting individual lesions; therefore, their usefulness is limited in more severe diseases when lesions tend to coalesce^36^. Furthermore, individual lesion counts were shown to differ across raters when compared to individuals with milder illnesses^43^.

## Treatment

Different methods for the management of HS are outlined in (Table 2).

**Table 2:** Treatment options for hidradenitis suppurativa.

Treatment	Advantages	Disadvantages
Lifestyle modification and general management Lifestyle modification^44–49^ Cessation of smoking Avoid rough clothing and tight underwear, jeans, belts, bras, and collars and opt for loose cotton fabrics Reducing body weight General management^2,6,29,50–53^ Pain management Wound care Psychological support	Valuable adjunctive options to conventional medical treatments. Useful in situations where prescription medications are unsuitable, e.g., immunosuppression or pregnancy. No side effects.	Insufficient as a solitary treatment strategy. It demands considerable time, dedication, and overcoming ingrained habits.
Topical treatment^2,54^ Topical clindamycin 1% Topical resorcinol 15% Skin cleansers	Simple to use and apply, with few systemic adverse effects, suitable for milder forms of HS.	Local side effects like skin irritation or allergic reactions. Not suitable for widespread illness.
Intralesional therapy^55–57^ Steroid (triamcinolone) injections	A safe, well-tolerated, and efficient method that is known to reduce discomfort, swelling and hasten the shrinkage of fistulous tracts and abscesses.	May result in skin atrophy, telengiectasia, and hypopigmentation. The risk of glycemic decompensation in diabetic patients requires caution.
Systemic antibiotics^5,58–62^ Oral tetracyclines (eg. doxycycline 50-100 mg twice daily) Oral clindamycin 300 mg twice daily plus oral rifampicin 600 mg daily for 12 weeks Moxifloxacin (400 mg daily), rifampicin (10 mg/kg daily), and metronidazole (500 mg thrice daily) for up to 12 weeks, with discontinuation of metronidazole after 6 weeks	A widely utilized and effective treatment option, oral antibiotics offer ease of administration and convenience, particularly for treating widespread or inaccessible areas. They can also be combined synergistically with other therapies like topicals or biologics for enhanced efficacy.	Side effects include gastointestinal disturbances, and sun sensitivity associated with tetracyclines; Clostridium difficile-associated diarrhea, rash, and hepatotoxicity linked to clindamycin; orange-tinged bodily fluids resulting from rifampin use; and methemoglobinemia and cyanosis of the lips associated with dapsone. Risk of leading to bacterial resistance
Biologics^63–68^ Adalimumab on Week 0 (160 mg S.C.), Week 2 (80 mg S.C.), and every week (40 mg S.C) for 12 weeks followed by assessment Secukinumab (300 mg weekly for 5 weeks, then every 4 weeks.)	Biologics specifically target the underlying inflammatory pathways involved in HS, offering high efficacy, long-term disease control, and improved overall well-being.	It requires careful monitoring due to infection, drug interaction risks, and the potential for malignancy, like lymphomas. Secukinumab has been associated with Crohn's disease.
Surgery^37,69–73^ Incision and Drainage Deroofing Excision Lasers	Offers significant advantages in severe cases of HS, particularly for addressing recurrent or persistent abscesses.	May lead to scarring, infection, bleeding, pain, wound dehiscence, and recurrence

HS, hidradenitis suppurativa.

### Lifestyle modification

Patients should avoid rough clothing and tight underwear, jeans, belts, bras, and collars, especially nonbreathable fabrics like polyester and nylon, as they can increase discomfort and lesion formation^44^, and opt for loose cotton fabrics^45^. Research indicates that 70–90% of HS patients smoke^46^. There are, however, conflicting findings about the relationship between smoking and the severity of HS^47^. Despite these findings, patients are suggested to avoid smoking since there is compelling evidence of the harmful pathophysiological effects of tobacco smoke and its byproducts on skin and HS lesions^48^. Reducing weight is highly recommended, as several studies have shown that a reduction in weight leads to major improvements^45,49^. Despite encouraging results, studies related to non-pharmacological therapies are inadequate. Nonetheless, they offer valuable adjunctive options to conventional medical treatments, particularly in situations where prescription medications are unsuitable, such as immunosuppression, or during pregnancy^45^.

### General management

#### Pain management

One major contributing factor to the reduced quality of life experienced by people with hidradenitis suppurativa (HS) is pain^6^. HS pain is similar to persistent post-traumatic headaches and more severe than blistering diseases, vulvar lichen sclerosis, vasculitis, and leg ulcers^6^.

To address acute pain, acetaminophen, nonsteroidal anti-inflammatory medications, and surgical techniques (such as incision and drainage of inflammatory nodules) are recommended^50^. Corticosteroids injections alone or with 1% xylocaine can be used to treat inflammatory nodules^50^.

Management of chronic pain should be based on the WHO pain ladder^29^. Nonsteroidal anti-inflammatory medications, pregabalin, gabapentin, venlafaxine, duloxetine, and, in more complex cases, judicious use of antidepressants may also be used for chronic pain^6^.

For severe pain, short-acting opioid analgesics can be prescribed individually and cautiously^29^.

#### Wound care

In a study of 50 dermatologists investigating wound care counseling in HS, the most generally prescribed dressings were abdominal pads (80%), gauze (76%), and pantyliners or menstrual pads (52%)^51^. The suggested methods for managing flares at home were Epsom salt baths (18%), zinc oxide lotion (28%), warm compresses (76%), bleach baths (50%), and Vicks VapoRub (12%)^51^. Warm compresses, followed by bleach baths and Epsom salt baths, had the largest proportion of dermatologists rating the product as outstanding for flares^51^.

#### Psychological support

Patients with HS experience severe psychological effects as a result of the discomfort and foul-smelling discharge from the lesions^2^. Group psychotherapy could be beneficial for these patients^52^. Psychological support for HS patients is critical and should be included in the disease management plan^52^.

### Topical therapy

Topical antibiotics, keratolytic agents, and skin cleansers are all part of the topical therapy for HS. Although there is no data to support the use of any particular skin cleanser, the use of zinc pyrithione, benzoyl peroxide, and chlorhexidine is supported by professional opinion^74^. The most commonly used topical antibiotic for HS is clindamycin 1%. Topical clindamycin 1% twice daily for 12 weeks is the first line of management for less severe stages of HS (Hurley I–II), with its effect comparable to oral tetracyclines^2^. Another topical agent used in HS is resorcinol 15%, a chemical peeling agent with keratolytic and anti-inflammatory properties^75^. A retrospective analysis of 134 individuals with mild-to-moderate HS (Hurley stages I and II) discovered that the response to 15% resorcinol outperformed the response to topical clindamycin 1% in terms of clinical response and disease-free survival^54^. The same study suggested that topical resorcinol 15% may be a useful substitute for clindamycin, reducing antibiotic usage and the development of antibiotic resistance^54^.

### Systemic antibiotics

Systemic antibiotics are a commonly utilized therapeutic strategy for HS and are indicated in every published HS treatment guideline^58–60^. Oral tetracyclines (e.g. doxycycline 50–100 mg twice daily) are the first-line treatment for milder diseases^5^. That said, it is believed that the anti-inflammatory rather than the antibacterial properties of tetracyclines and other antibiotics provide their benefits in HS^58,59^.

A combination of systemic antibiotics are also used for the management of HS. A study concluded that the combination of oral clindamycin 300 mg twice daily with oral rifampicin 600 mg daily for 12 weeks was effective and tolerable for the majority of the patients with HS^61^. Certain antibiotic regimens have been proposed involving multiple antibacterial drugs, including moxifloxacin (400 mg daily), rifampicin (10 mg/kg daily), and metronidazole (500 mg thrice daily) for up to 12 weeks, with discontinuation of metronidazole after 6 weeks for HS not responding to therapy^60^.

### Other systemic therapy

#### Hormonal therapy

Anti-androgenic medication has been shown to enhance clinical outcomes in female HS patients^76^. Hormonal agents, such as estrogen-containing oral contraceptives, spironolactone, cyproterone acetate, metformin, and finasteride, can be used as monotherapy for mild-to-moderate HS or in combination with other medications for more severe disease^74,76^. In a retrospective analysis of 20 female HS patients treated with spironolactone 100 to 150 mg daily, 85% (17/20) showed improvement^77^. Four global case reports comprising 13 patients investigated the efficacy of finasteride (1.25–10 mg/d) for HS, collectively demonstrating the beneficial effects of finasteride in treating HS^78^. The link between HS and PCOS is well established, with some authors even proposing that all female patients presenting with HS be tested for underlying PCOS and insulin resistance^79^. Recently, several studies have been published on the advantages of metformin in HS, which have been related to its anti-inflammatory properties, effects on insulin sensitivity and glucose metabolism, and influence on hormones like androgens^80,81^. Metformin may be recommended for people who have concomitant diabetes or polycystic ovarian syndrome^62^. An uncontrolled prospective study comprising 25 patients with HS, the great majority of whom were female and some had PCOS, found considerable clinical effectiveness of metformin (500 mg 2–3 times a day) in treating HS^79^. Women whose HS worsens with the menstrual cycle and have a shorter disease course may benefit the most from OCP therapy. It has been reported that OCPs may improve the number of abscesses and inflammatory nodules (AN) in women with HS^82^.

#### Oral isotretinoin

Isotretinoin is only recommended as a second or third line of treatment, or for people who have concomitant acne^5^. In a retrospective study with 39 HS participants, 14 patients (or 35.9%) indicated that isotretinoin had a positive effect^83^. Another retrospective analysis found a 68% isotretinoin response rate in their HS patient population. According to the same study, those who responded to isotretinoin therapy in any way were more likely to be female, younger, weigh less, and have acne^84^.

#### Cyclosporine

It has been suggested that cyclosporine might be used to treat recalcitrant HS^85^. According to a case study, 9 out of 18 severe HS patients responded well to cyclosporine treatment^86^.

#### Methotrexate

According to published studies, methotrexate may not be an effective treatment for HS^87^. However, a retrospective study suggests that methotrexate may be an effective therapy in a certain subset of patients with HS, particularly older people with lower body mass indices, although it seems ineffective in patients treated in combination with biological therapies^88^.

#### Oral steroid therapy

Oral corticosteroids are helpful as a low-dose adjuvant with biologics and other immunomodulating drugs, and they are known to offer rapid relief in acute flare-ups of HS^59^. High-dose systemic corticosteroids have been shown to be useful in treating HS; however, the benefit was short-lived after tapering of the dose. To establish long-term control and minimize unwanted side effects, treating HS with low-dose systemic steroids over time is an alternate treatment option^89^.

### Intralesional steroid therapy

Intralesional steroids are beneficial in acute flares^55^. Intralesional triamcinolone injection into inflammatory HS lesions results in a considerable decrease in erythema, edema, suppuration, and nodule size^62^.

When using intralesional steroids, a prospective study with 247 lesions that evaluated ultrasound-guided intralesional steroids injections in HS patients found that 81.1% (30/37) of nodules, 72% (108/150) of abscesses, and 53.3% (32/60) of draining fistulas responded to the steroid injections^55^. In another retrospective, multi-center study involving HS patients receiving intralesional corticosteroid injection, 95 lesions (70.37%) exhibited a complete response, 34 showed a partial response (25.19%), and 6 (4.44%) showed no response at all^56^. However, according to another research, there was no statistically significant difference identified between different doses of triamcinolone and normal saline for the treatment of acute HS lesions over a period of 14 days, suggesting that steroid injections may not be as beneficial for managing acute HS as is commonly believed^57^. Despite there being no statistically significant difference in the effectiveness of triamcinolone injections compared to normal saline, the average patients did report experiencing some degree of improvement. The authors suggested the possibility of relief being linked to the act of puncturing a lesion or introducing an external solution^57^.

### Biologics

Adalimumab, a TNF-α inhibitor, is the first medication approved by the European Medicines Agency (EMA) and the Food and Drug Administration (FDA) for moderate-to-severe HS^63^. Adalimumab is prescribed in HS at a dosage of 160 mg on Week 0, 80 mg on Week 2, and then a 40 mg injection every week^64,65^. Reviewing the continuation of adalimumab medication is necessary if a patient does not exhibit improvement by week 12. Using a topical antiseptic wash on HS lesions every day is advised throughout adalimumab therapy, and antibiotics may be continued if needed^64^.

Until 31 October 2023, adalimumab stood as the sole FDA-approved biologic drug for HS until secukinumab was granted FDA approval for the same condition^65,66^. Secukinumab is a biological drug that preferentially targets interleukin-17 (IL-17)^65^. In a multi-center retrospective study, 31 patients who had failed or were contraindicated for at least one anti-TNF-alpha were treated with secukinumab^67^. In this trial, 41% of patients at week 28 attained the Hidradenitis Suppurativa Clinical Response Score (HiSCR), with the sole adverse effect noted being a facial eruption resembling acne^67^.

Another study with twenty HS patients used subcutaneous injections of secukinumab 300 mg once a week for five weeks, then once every four weeks for a total of sixteen weeks. Seventy-five percent of patients (15/20) had a satisfactory HiSCR response after 16 weeks of treatment, whereas two individuals developed Crohn’s disease (CD) after three and five months of therapy^68^.

Additional biologics are under investigation for their potential application in treating HS, such as IL-1 inhibitors (anakinra^90,91^, canakinumab^92,93^), TNF-α inhibitors (infliximab^94–96^, etanercept^97^), IL-17 inhibitors (bimekizumab^98^), IL-23 inhibitors (guselkumab^99,100^, risankizumab^101,102^), IL-12 and IL-23 inhibitor (ustekinumab^103^), phosphodiesterase-4 inhibitors (apremilast^104^), Janus kinase 1 inhibitor (INCB054707^105^), and chimeric monoclonal antibody against the CD20 protein (rituximab^106,107^).

### Surgery

Surgery is frequently necessary and quite prevalent in HS patients since nonsurgical alternatives often result in an unsatisfactory outcome^37^. The location and extent of HS, as well as whether the excision is sufficient, are all important factors in the surgical process^69^. Local excision of single lesions is only suggested for limited, well-defined lesions of Hurley I and II^37^.

For patients presenting with acute, painful, fluctuant abscesses, incision and drainage (I&D) is a beneficial technique^70^. While its high recurrence rate makes it unsuitable for long-term practice, it is helpful in treating acute pain and suffering^70^. For large chronic lesions, an extensive excision, CO_2_, or electrosurgical excision (with or without repair) is recommended^29,108^.

Deroofing, a minimally invasive procedure that can be performed under local anesthesia, involves stripping the “roof” from abscesses or sinus tracts to expose lesion floors in Hurley stage I or II hidradenitis suppurativa^71^. Deroofing is a surgery option that provides cosmetically acceptable results and prevents contractures^71^. Many techniques, including primary suturing, skin grafting, flap reconstruction, and secondary healing, have been employed to promote healing of the postsurgical defects without wound contracture and postsurgical complications^72^. Less invasive techniques including platelet-enriched plasma, dermal replacements, and vacuum-assisted closure (VAC treatment) provide a significant boost to the reconstructive strategy^70^. Continuing medical treatment throughout the perioperative period may be advantageous and reduce the risk of postoperative complications^29^.

Lasers, such as Nd:YAG or CO_2_, are emerging as potential therapy techniques for hidradenitis suppurativa since they provide focused tissue destruction while reducing lesion burden with little invasiveness^73^. While studies have shown promising results for CO_2_ laser therapy, further study is needed to determine its long-term usefulness^71,73^.

## Hyperhidrosis treatments in HS

Hyperhidrosis reduces the quality of life for people with hidradentitis suppurativa by aggravating odor and pruritus^109,110^. An age-adjusted and gender-adjusted multivariable logistic regression revealed a 3.61-fold (95% CI 2.83–4.61, *P*<0.0001) higher risk of hyperhidrosis in HS patients, indicating a link between hyperhidrosis and HS^111^. Treatment of hyperhidrosis in locations where individuals have HS lesions may help both illnesses^109^. Hyperhidrosis treatments tested in HS patients include botulinum toxin A (BTX-A), botulinum toxin B (BTX-B), suction-curettage, 1450 nm diode laser, and microwave-based energy device (MED)^109^. A systematic review found that treatments for hyperhidrosis, including BTX, may be helpful in the treatment of HS, while MEDs may be harmful^109^.

## Strengths and limitations

This literature review has several strengths, such as predominantly comprising of papers published in the recent years. It provides an overview of the diagnosis and treatment options for HS and their respective advantages and disadvantages. However, this literature review has some limitations. Firstly, it does not provide the same level of evidence as randomized controlled trials (RCTs) or meta-analyses. Furthermore, because this review is limited to papers published in English it may miss valuable insights from studies published in other languages.

## Conclusion

This literature review gives an overview of both the assessment tools and the current therapeutic strategies for HS. Despite important advances made in recent studies, the pathophysiology of HS remains unclear. Multiple factors including lifestyle, genetic and hormonal factors are linked to the development of HS. Although various modalities of treatment exist for HS, such as lifestyle modifications, topical and systemic antibiotics, and surgical interventions, the evidence supporting their efficacy is quite limited. It is essential for patients with HS to avoid trigger factors such as smoking and obesity, wear loose-fitting cotton clothing, and apply topical therapy. In cases where a patient shows inefficacy of topical and oral antibiotics, consideration of intralesional steroids, systemic steroids, oral retinoids, hormone therapy, biologics, or other treatments is given. Surgical management may also be required for severe cases. Because no medication works universally for all patients, those with HS require personalized treatment strategies and multidisciplinary collaboration from dermatologists, surgeons, pain specialists and mental health professionals. Future research should focus on broadening the current understanding of HS pathogenesis and assessing new treatments in clinical trials.

## Ethical approval

Ethics approval was not required for this review.

## Consent

Informed consent was not required for this review.

## Source of funding

Funding was not received for this study.

## Author contribution

A.P.: concept, manuscript preparation, edit and review.

## Conflicts of interest disclosure

The author of this paper has no conflicting interests.

## Research registration unique identifying number (UIN)

Not applicable.

## Guarantor

Archana Pandey.

## Data availability statement

Data sharing is not applicable to this article.

## Provenance and peer review

Not commissioned, externally peer-reviewed.
